# ﻿Resurrection of *Perilimnastes* (Sonerileae, Melastomataceae) with description of a new species *P.nana*

**DOI:** 10.3897/phytokeys.238.116168

**Published:** 2024-02-01

**Authors:** Ying Liu, Jin-Hong Dai, Qi-Yuan Zhuang, Chun-Yu Zou, Kai-Nan Ma

**Affiliations:** 1 School of Ecology, Sun Yat-sen University, Shenzhen 518107, China; 2 State Key Laboratory of Biocontrol and Guangdong Key Laboratory of Plant Resources, Sun Yat-sen University, No. 135, Xin-Gang-Xi Road, Guangzhou 510275, China; 3 School of Life Sciences, Sun Yat-sen University, Guangzhou 510275, China; 4 Guangxi Institute of Botany, Guangxi Zhuang Autonomous Region and the Chinese Academy of Sciences, Guilin 541006, China

**Keywords:** Melastomataceae, *Perilimnastes*, *Phyllagathis*, taxonomy

## Abstract

Recent research has indicated that the *Phyllagathis* (raphides) clade (Sonerileae, Melastomataceae) is only distantly related to the type of *Phyllagathis* and should be separated as a distinct genus. Phylogeny of this clade is here reconstructed with expanded taxon sampling. Four strongly supported subclades have been identified. The possible affinities of taxa that were not sampled in the analysis are discussed, based on morphological data. *Perilimnastes* is resurrected as the generic name of the *Phyllagathis* (raphides) clade. A generic description, colour figures, map of distribution, a list of included species and a key are provided for *Perilimnastes*. Fifteen new combinations are made plus the description of a new species. As interpreted here, *Perilimnastes* consists of twenty species and two varieties.

## ﻿Introduction

**Figure 1.**
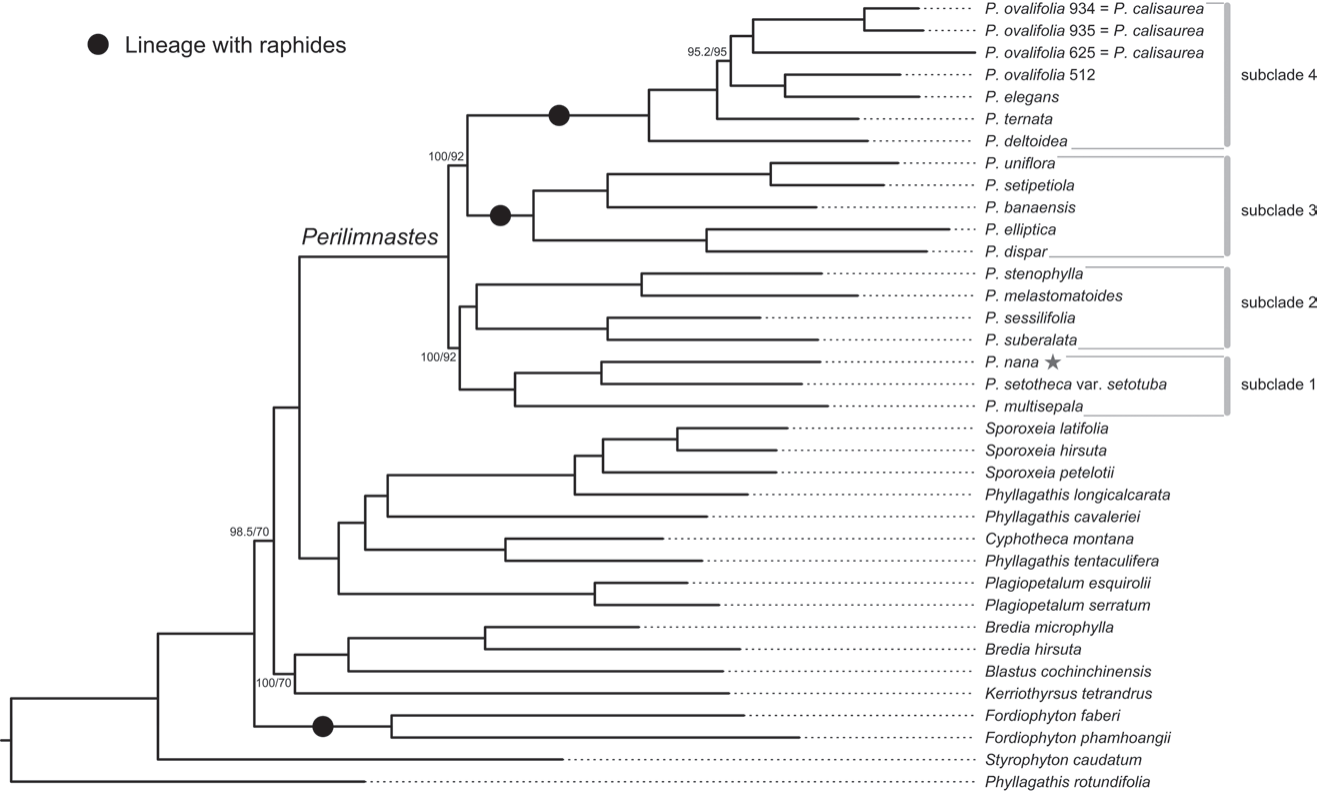
The partitioned Maximum Likelihood (ML) phylogenetic tree inferred from the genomic SNP dataset using IQ-TREE, showing the four subclades within *Perilimnastes* [*Phyllagathis* (raphide) clade]. For the nodes without full support, values from SH-aLRT test (left) and ultrafast bootstrap (right) are given at the nodes. The new species is indicated with a star. Lineages with raphides are noted with solid circles.

The genus *Perilimnastes* Ridl. was initially established based on *P.fruticosa* (Ridl.) Ridl. (Ridley 1918, 1922). Nayar (1974) followed Ridley’s concept of *Perilimnastes* and described a second species in the genus, namely *P.rupicola* M.P.Nayar. The two species show clear similarities in the morphology of leaves, calyx lobes, stamens, and capsules. However, subsequent authors did not recognise *Perilimnastes* (Maxwell 1982, 1989; Cellinese 2002, 2003). Both species are currently treated in a broadly defined *Phyllagathis* Blume. Previous molecular phylogenetic studies have revealed the polyphyletic nature of *Phyllagathis* (Zeng et al. 2016; Zhou et al. 2018; Zhou et al. 2019a, b, c; Liu et al. 2022; Zhou et al. 2022). The species currently treated under *Phyllagathis* were found to be nested within 17 lineages of Asian Sonerileae (Liu et al. 2022; Zhou et al. 2022). Although *P.fruticosa*, the generic type of *Perilimnastes*, was not sampled in these studies, species that are quite similar to it were identified as belonging to the *Phyllagathis* (raphides) clade. Members of this clade are often shrubs or shrublets with cuneate to rounded leaf bases, umbellate or cymose inflorescences (sometimes reduced to a single flower), isomorphic stamens, dorsally spurred connectives, crowned capsules, horned placental column and thready placentas. Some of them (Fig. 1) are also characterised by the presence of raphide crystals in various parts of the plant. Based on these diagnostic features as well as strong resemblance between sampled and unsampled species, Zhou et al. (2022) estimated that the *Phyllagathis* (raphides) clade might contain 20 species in southernmost China, Vietnam, the Malay Peninsula and Borneo. This clade should be removed from *Phyllagathis* and treated as a distinct genus, since it is only remotely related to the type of *Phyllagathis*. As a result, *Perilimnastes* should be re-instated as the generic name (Zhou et al. 2022).

This work aims to formalize the taxonomic treatment of the *Phyllagathis* (raphides) clade. To this end, we reconstructed the phylogeny of this clade with expanded taxon sampling, using a nuclear genomic dataset assembled by mapping the genome resequencing reads to the draft genome of *Brediahirsuta* Blume. We also discussed putative affinities based on morphological data for species that were not sampled in the phylogenetic analysis. *Perilimnastes* is resurrected as the generic name of the *Phyllagathis* (raphides) clade. A generic description, colour figures, map of distribution, a list of included species and a key are provided for *Perilimnastes*. Fifteen new combinations are made plus the description of a new species from southern China. *Perilimnastes*, as we here delimit it, now consists of twenty species and two varieties.

## ﻿Methods

### ﻿Phylogenetic reconstruction

For phylogenetic reconstruction of the *Phyllagathis* (raphides) clade, ingroups and outgroup were selected according to the genomic tree of Sonerileae (Zhou et al. 2022). We sampled 36 accessions from Sonerileae, including 16 species from the *Phyllagathis* (raphides) clade, as well as species of *Phyllagathis* [including the generic type *P.rotundifolia* (Jack) Blume], *Styrophyton* S.Y.Hu, *Bredia* Blume, *Fordiophyton* Stapf, *Blastus* Lour., *Kerriothyrsus* C.Hansen, *Cyphotheca* Diels, *Plagiopetalum* Rehder and *Sporoxeia* W.W.Sm. (Suppl. material 1: table S1).

For DNA extraction, library preparation, whole genome resequencing and quality control of the raw reads, methods employed in this study followed the protocols outlined in Zhou et al. (2022). The genomic single nucleotide polymorphism (SNP) dataset was assembled by mapping the genome resequencing data to the draft genome of *Brediahirsuta*, which can be accessed at https://doi.org/10.17632/s85vv6yyjs.1. High-quality reads were mapped to the reference genome using BWA-MEM (Li and Durbin 2010). SNPs and short insertions/deletions (InDels) were identified using HaplotypeCaller in GATK v.4.1.8.1 (McKenna et al. 2010) under the GVCF mode for each sample separately. Next, we conducted hard filtering to minimise false positives by applying the following parameters: (1) QUAL < 30.0; (2) DP < 15.0; (3) QD < 2.0; (4) FS > 60.0; (5) MQ < 50.0; (6) SOR > 3.0; (7) MQRankSum < -12.5; (8) ReadPosRankSum < -8.0; (9) InbreedingCoeff < -0.5. VCFtools v.0.1.16 (Danecek et al. 2011) is used to exclude SNPs with a missing rate exceeding 15% and those with minor allele frequencies (MAF) below 0.05. The SNPs obtained were pruned, based on their linkage disequilibrium (LD) patterns using the –indep-pairwise option in PLINK (Purcell et al. 2007). Only one SNP was retained for each SNP pair with an *r*^2^ value above 0.5 within a sliding window of 50-SNPs (advanced by 5 SNPs each).

Maximum Likelihood analysis of the genomic dataset was performed using a partitioned approach in IQ-TREE v.2.0.3 (Nguyen et al. 2015). *Phyllagathisrotundifolia* was designated as the outgroup taxon. The selection of best fitting substitution model was conducted using ModelFinder (Kalyaanamoorthy et al. 2017) based on the Bayesian Information Criterion (BIC). The genomic dataset was partitioned into bins of equal length, each containing 2,000 SNPs. TVMe+ASC+R2 was selected as the best fitting substitution model for all partitions. Node support was accessed using 1000 replicates of the UFBS and SH-aLRT test.

### ﻿Morphological comparison

Morphological data were obtained through fieldwork, herbarium records, literature survey and observation of living plants in the facilities of Sun Yat-sen University. We examined specimens or their high-resolution photos of the relevant species from the following herbaria: A, BM, C, E, G, GXMI, IBSC, IBK, K, KUN, NY, P, PE, SYS and US. Species delimitation mainly followed Chen (1984a), Hansen (1992), Cellinese (2002, 2003) and Chen and Renner (2007).

## ﻿Results and discussion

### ﻿Phylogenetic relationships

After SNP filtering and pruning, the genomic dataset contained 2,412,522 SNPs, 1,667,363 of which were parsimony informative, with 26.46% of missing data (available at http://doi.org/10.17632/g9yjn97kns.2). The partitioned genomic ML tree was presented in Fig. 1. All nodes in the tree received full support (SH-aLRT test = 100%, UFBS = 100%), except for five nodes (Fig. 1).

Four well-supported lineages were identified within the *Phyllagathis* (raphides) clade, but relationships amongst them were only moderately supported (SH-aLRT test = 100%, UFBS = 92%; SH-aLRT test = 100%, UFBS = 92%). Subclade 1 contains *Perilimnastesmultisepala* J.H.Dai, T.V.Do & Ying Liu from central Vietnam, *Phyllagathissetotheca* H.L.Li from southern China and a new species from Guangdong, China, namely *Perilimnastesnana* C.Y.Zou & Ying Liu. The three species are characterised by large flowers (> 20 mm in diameter), large anthers (> 8 mm long) and the presence of druses (instead of raphides). Subclade 2 consists of two species from Hainan Island, China [*Phyllagathisstenophylla* (Merr. & Chun) H.L.Li and *P.melastomatoides* (Merr. & Chun) W.C.Ko] and two from central Vietnam (*P.suberalata* C.Hansen and *P.sessilifolia* C.Hansen). Species in this subclade varied in the morphology of leaves and flowers, but they all have druses and yellow connectives that produced into a collar at the anther base. Subclade 3 comprises two species from Borneo [*Phyllagathisdispar* (Cogn.) C.Hansen and *P.elliptica* Stapf] and three newly-published species from central and southern Vietnam (*Perilimnastessetipetiola* J.H.Dai, T.V.Do & Ying Liu, *P.uniflora* J.H.Dai, T.V.Do & Ying Liu and *P.banaensis* J.H.Dai, T.V.Do & Ying Liu). These species are morphologically quite different, yet all of them have raphide crystals, somewhat elliptic leaf blade and at least some have terminal and axillary umbels with very short or no peduncles. Subclade 4 consists of five taxa mainly distributed in southern China, viz. *Phyllagathisdeltoidea* C.Chen, *P.elegans* Hai L.Chen, Yan Liu & Ying Liu, *P.ternata* C.Chen, *P.ovalifolia* H.L.Li and *P.calisaurea* C.Chen (currently synonymised under *P.ovalifolia*). These species have raphides and share obvious similarities in the inflorescences with 1–3.5 cm long peduncles and purple anthers with a short dorsal spur and without ventral appendages. Zhou et al. (2022) found that the crystal type exhibits the lowest level of homoplasy amongst 14 characters they tested. The shift from druses to raphides took place on only three occasions within Asian Sonerileae (Zhou et al. 2022), one in *Fordiophyton* and two in two subclades of the *Phyllagathis* (raphides) clade. The presence of raphides, therefore, is a useful diagnostic character for these lineages.

### ﻿Species without molecular data

*Perilimnastesfruticosa*, *Phyllagathisguillauminii* H.L.Li, *Phyllagathisbrookei* M.P.Nayar and *Perilimnastesrupicola* M.P.Nayar, four putative members of the *Phyllagathis* (raphides) clade, have never been included in phylogenetic studies. Nevertheless, they can be easily referred to specific lineages within this clade, based on compelling morphological evidence. *Perilimnastesfruticosa* from the Malay Peninsula closely resembles *P.multisepala* from subclade 1 and *P.stenophylla* and *P.suberalata* from subclade 2. The four species are shrubs characterised by somewhat oblong-lanceolate, 3-veined leaf blades, few-flowered inflorescences, narrow calyx lobes and the presence of druses. Moreover, they grow in similar habitats, specifically on rocks along streams in dense forests. *Perilimnastesfruticosa* is possibly a member of subclade 1 or subclade 2. Raphides have been found in the tissues of *P.guillauminii* (southern Vietnam), *P.brookei* (Borneo) and *P.rupicola* (Borneo). The three species can be confidently referred to subclade 3 since all Vietnamese and Bornean species with raphides were consistently recovered as members of this subclade in phylogenetic analyses (Zhou et al. 2022; this study). *Phyllagathisguillauminii* resembles *P.uniflora* from subclade 3 in 3-veined leaves with cuneate base and somewhat acuminate apex and narrow calyx lobes. The close relationships amongst *P.dispar*, *P.elliptica*, *P.brookei* and *P.rupicola* had been proposed by Cellinese (2003). Their caulescent and erect stems, small leaves, few-flowered umbels, as well as crystal type make them a distinct group that is morphologically very different from other Bornean species treated under *Phyllagathis* (Cellinese 2003).

Another species, *P.marumiaetricha* (Guillaumin) C.Hansen, was listed as a putative member of the *Phyllagathis* (raphides) clade by Zhou et al. (2022). It resembles *P.setotheca* from subclade 1 in the inflorescences with large basal bracts, petals and flowers. However, the huge leaves, distinctive hypanthial emergences and the peculiar sepals of this species readily distinguish it from members of the *Phyllagathis* (raphides) clade. As its generic affiliation remains to be further tested, no taxonomic treatment is proposed here.

## ﻿Conclusion

Molecular phylogenetic data and morphological evidence support the *Phyllagathis* (raphides) clade as a distinct lineage encompassing species distributed in southernmost China, Vietnam, the Malay Peninsula and Borneo. *Perilimnastes* is, therefore, resurrected below as the generic name for this clade. For a comparison of *Perilimnastes* [the *Phyllagathis* (raphides) clade] and other lineages of Asian Sonerileae, please see table S9 in Zhou et al. (2022).

## ﻿Taxonomy

### ﻿ Perilimnastes

Taxon classificationPlantaeMyrtalesMelastomataceae

Ridl., J. Straits Branch Roy. Asiat. Soc. 79: 70. 1918, emend. Ying Liu

26605EC3-7E86-50CB-A0DB-9ABF1C742866

#### Type.

*Perilimnastesfruticosa* (Ridl.) Ridl., J. Straits Branch Roy. Asiat. Soc. 79: 70, in obs. 1918; Ridley, Fl. Mal. Penins. 1: 773. 1922.

#### Description.

**Figure 2.**
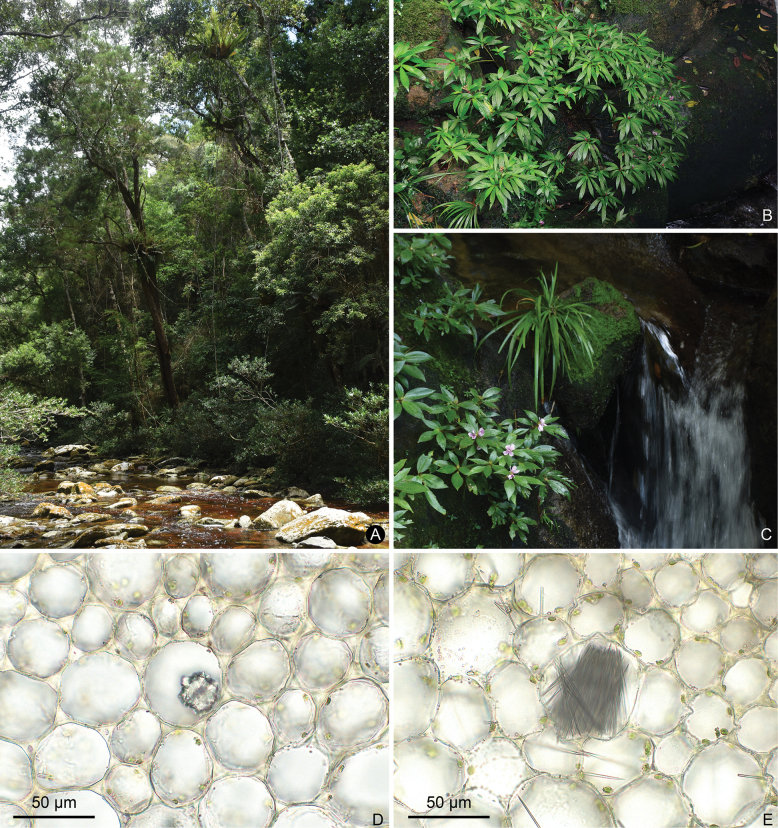
Habitat (**A–C**) and crystal type (**D, E**) of *Perilimnastes***A***P.stenophylla***B***P.elegans***C***P.melastomatoides***D** druses of *P.multisepala***E** raphides of *P.elegans*. Scale bars: 50 μm (**D, E**).

**Figure 3.**
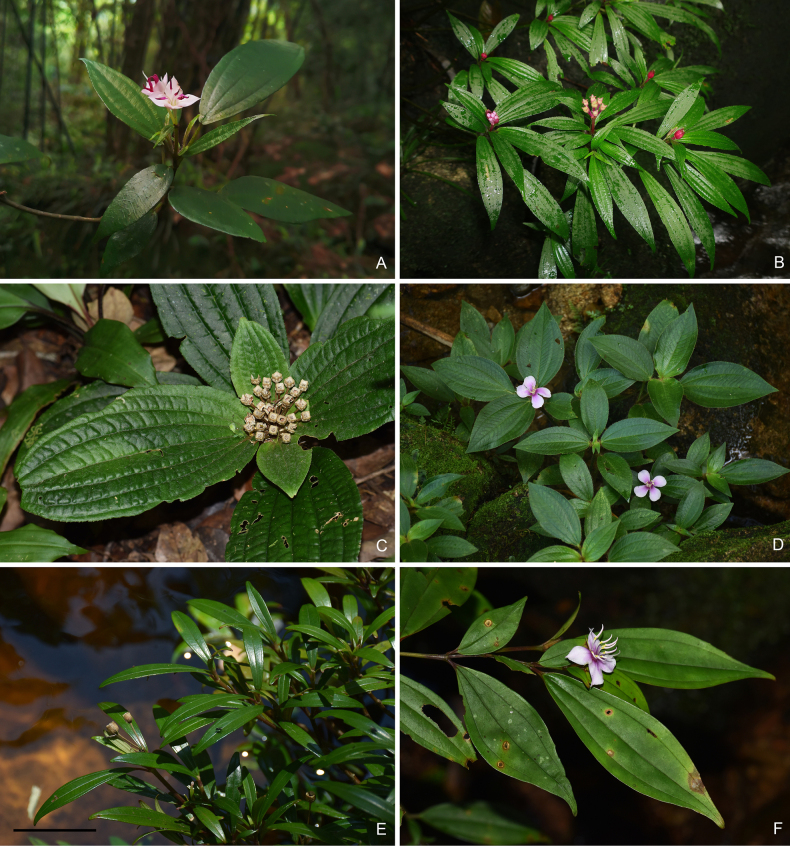
Flowering/fruiting branches of *Perilimnastes***A***P.deltoidea***B***P.elegans***C***P.elliptica***D***P.melastomatoides***E***P.stenophylla***F***P.suberalata*.

**Figure 4.**
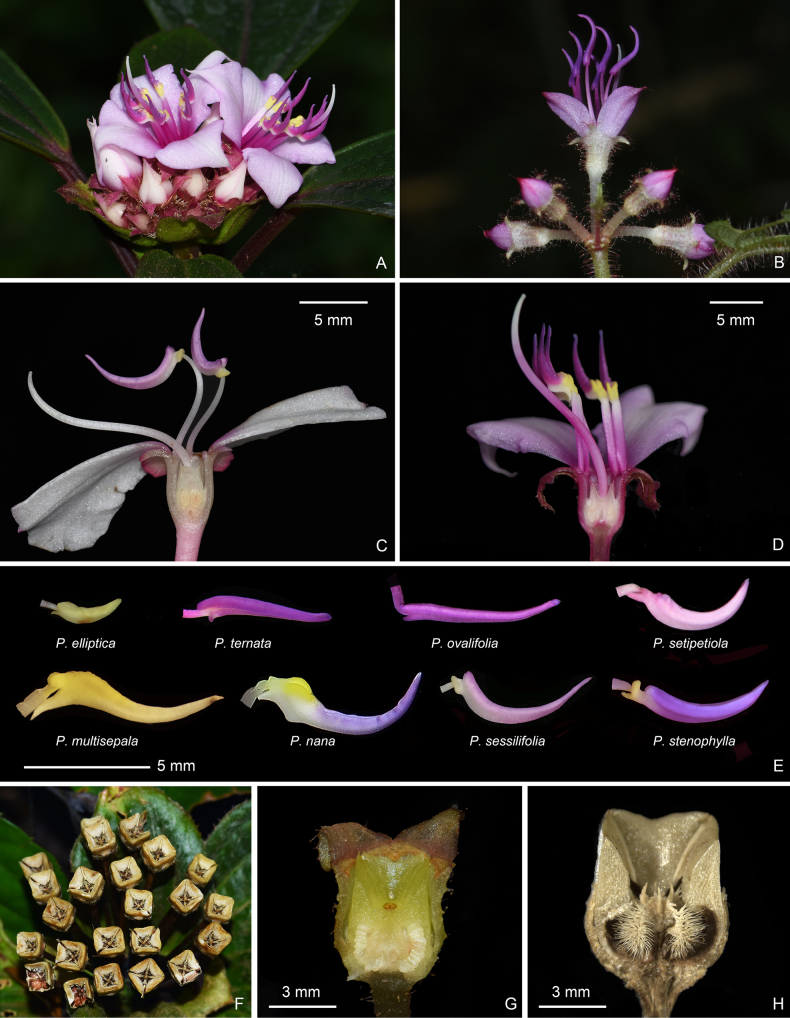
Inflorescence (**A, B**), longitudinal section of flower (**C, D**), anther morphology (**E**), infructescence (**F**), and longitudinal sections of young and old capsules (**G, H**) of *Perilimnastes***A***P.setotheca***B***P.ternata***C***P.sessilifolia***D***P.setotheca***E***P.elliptica*, *P.ternata*, *P.ovalifolia*, *P.setipetiola*, *P.multisepala*, *P.nana*, *P.sessilifolia* and *P.stenophylla* (from left to right and top to bottom) **F***P.elliptica***G***P.ovalifolia***H***P.ovalifolia*. Scale bars: 5 mm (**C–E**); 3 mm (**G, H**).

Erect shrubs, erect/ascending shrublets or caulescent herbs, sometimes with raphides in many parts. Stems terete, obtusely 4-sided or ribbed, with uniseriate or multiseriate, appressed or spreading hairs, rarely glabrous. Leaves opposite, equal, subequal or unequal in a pair, petiolate, rarely sessile (in *P.sessilifolia*); leaf blades elliptic, ovate, elliptic-lanceolate, obovate, oblanceolate or suborbicular, submembranous, papery or stiffly papery, 3–7-nerved, base cuneate, acute, rounded, subcordate to broadly cordate, margin entire or inconspicuously serrulate or denticulate. Inflorescences usually terminal (rarely axillary) umbels subtended by two or more bracts, many- to few-flowered, sometimes reduced to a single flower. Flowers 4-merous; hypanthia ± campanulate, cup-shaped or funnel-shaped; calyx lobes triangular, ± attenuate to ligulate or linear; petals white, pink or purplish, obovate, ovate, oblong, or elliptic, more or less oblique, apex acute or acuminate; stamens 8, equal or subequal; anthers isomorphic, yellow, pinkish or purplish, narrowly ovate to lanceolate, curved to ventral side, connectives ventrally inappendiculate and dorsally spurred, or basally forming a collar with two ventral auricles/lobes/ridges and a dorsal spur; ovary half inferior, ovoid, 4-celled, crown of four partly or fully connate lobes; style filiform. Old capsule cup-shaped, campanulate, quadrangular, crown persistent and enlarged, enclosing an obpyramidal space; placental column 4-horned; placentas thready. Seeds numerous, minute, cuneate. (Figs 2–4)

#### Distribution.

**Figure 5.**
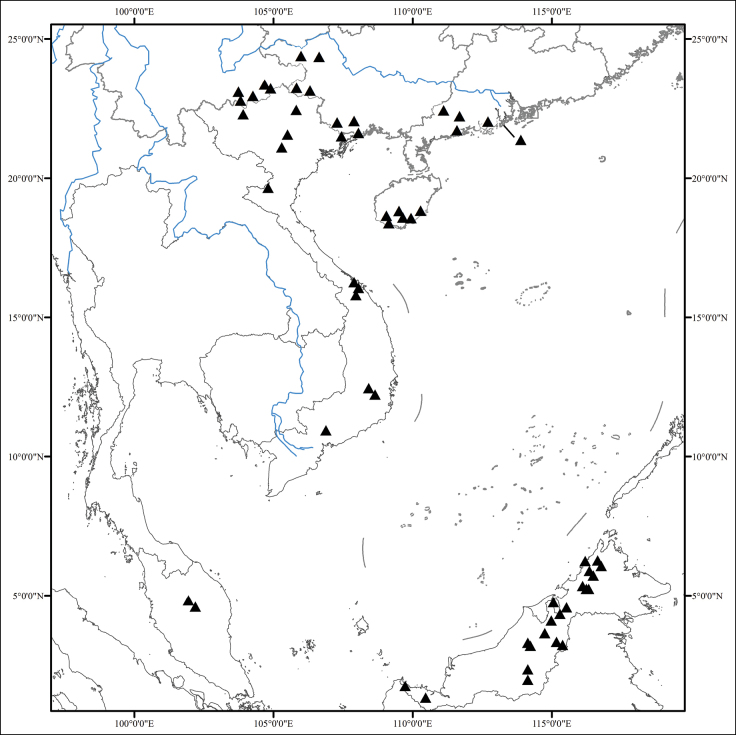
Distribution of *Perilimnastes*.

Twenty species and two varieties, eight species (seven endemic) and two varieties in southernmost China (Guangdong, Guangxi, Hainan, Yunnan), eight (seven endemic) in Vietnam, one on the Malay Peninsula and four in Borneo (Fig. 5).

##### ﻿Species included in *Perilimnastes*

### ﻿ Perilimnastes banaensis

Taxon classificationPlantaeMyrtalesMelastomataceae

J.H.Dai, T.V.Do & Ying Liu, PhytoKeys 235: 14. 2023.

A639C1E2-3989-5B33-A5BF-87BBA5CFF23F

#### Type.

Vietnam. Đà Nẵng: Hòa Ninh, Ba Na Hills, 1,360 m elevation, in forests on damp slopes near steam, 22 Nov 2019, Jin-hong Dai and Ying Liu 813 (holotype: PE; isotypes: A, SYS, VNMN).

### ﻿ Perilimnastes brookei

Taxon classificationPlantaeMyrtalesMelastomataceae

(M.P.Nayar) Ying Liu comb. nov.

26D22097-A4AB-5529-8A68-64B1E2D69FCE

urn:lsid:ipni.org:names:77335505-1

Phyllagathis brookei M.P.Nayar, J. Jap. Bot. 51(8): 232. 1976 (Basionym). Type: Malaysia. Sarawak: Bilengki, Bakelalan, 16 Aug 1955, W.M.A Brooke 10416 (holotype: BM! [BM000019481]).

### ﻿ Perilimnastes deltoidea

Taxon classificationPlantaeMyrtalesMelastomataceae

(C.Chen) Ying Liu comb. nov.

C1709B39-040C-53BE-AF65-32D44231B9DD

urn:lsid:ipni.org:names:77335506-1

Phyllagathis deltoidea C.Chen, Bull. Bot. Res., Harbin 4(3): 48. 1984 [“deltoda”] (Basionym). Type: China. Guangxi: Ningming, Mingjiang, Aidian, Gongmushan, 4,000 feet elev., 16 Dec 1935, H.H.Soo 68119 (holotype: IBSC! [IBSC0003993]; isotypes: IBK! [IBK00190675, IBK00190676]).

### ﻿ Perilimnastes dispar

Taxon classificationPlantaeMyrtalesMelastomataceae

(Cogn.) Ying Liu comb. nov.

4024FFA7-F4BC-52A5-A663-2B523516ABF8

urn:lsid:ipni.org:names:77335507-1

Anerincleistus dispar Cogn. ex Boerl., Handl. Fl. Ned. Ind. (Boerlage) i. 2: 531. 1890; et in DC. Monog. Phan. vii: 479. 1891 (Basionym). Type: Malaysia. Sarawak: O.Beccari 2400 (holotype: FI; isotypes: K! [K000867722], P! [P02274765]).
Phyllagathis dispar (Cogn.) C.Hansen, Nordic J. Bot. 2(6): 559. 1983.
Phyllagathis uniflora Stapf, Hooker’s Icon. Pl. 23: t. 2280. 1894. Type: Malaysia. Sabah: Kinabalu, 1892, G.D.Haviland 1172 (holotype: K! [K000867723]; isotypes: K! [K000867724], SAR, SING).
Phyllagathis uniflora var. longiloba M.P.Nayar, J. Jap. Bot. 51(8): 233. 1976. Type: Malaysia. Sabah: Kinabalu, Ulu Langanani, Sungei Mamut, 4,500 feet elev., 8 Aug 1961 W.L.Chew, E.J.H.Corner, and A.Stainton 1262 (holotype: K! [K000867721]; isotypes: L, SAR, SING).

### ﻿ Perilimnastes elegans

Taxon classificationPlantaeMyrtalesMelastomataceae

(Hai L.Chen, Yan Liu & Ying Liu) Ying Liu comb. nov.

9476CD1D-D894-5F79-B909-4AE166448DB8

urn:lsid:ipni.org:names:77335508-1

Phyllagathis elegans Hai L.Chen, Yan Liu & Ying Liu, Phytotaxa 509(2): 225. 2021 (Basionym). Type: China. Guangxi: Dongxing County, Ma-lu Town, Ping-feng Village, Yuan-ling, Shi-men Valley, on rocks and along grassy streamside in forests, 400–450 m elev., 9 Sept 2020, H.L.Chen, S.Y.Nong, and J.Q.Huang JHC343 (holotype: IBK!; isotypes: A!, IBSC!, PE!)

### ﻿ Perilimnastes elliptica

Taxon classificationPlantaeMyrtalesMelastomataceae

(Stapf) Ying Liu comb. nov.

A4546365-478A-5A22-8CFC-E0E3F86160F9

urn:lsid:ipni.org:names:77335509-1

Phyllagathis elliptica Stapf, Hooker’s Icon. Pl. 23: t. 2279. 1894 (Basionym). Type: Malaysia. Sabah: Kinabalu, G.D.Haviland 1286 (lectotype, designated by Cellinese [2003]: K! [K000867720]).

### ﻿ Perilimnastes fruticosa

Taxon classificationPlantaeMyrtalesMelastomataceae

(Ridl.) Ridl., J. Straits Branch Roy. Asiat. Soc. 79: 70, in obs. 1918; Ridley, Fl. Mal. Penins. 1: 773. 1922.

D9EB8938-8F25-54AC-974F-21E79E2E9E29

Anerincleistus fruticosus Ridl., J. Linn. Soc., Bot. xxxviii. 309. 1908 (Basionym). Type: Malaysia. Pahang: Gunong Tahan, 2 Jul 1905, L.Wray and H.C.Robinson 5453 (lectotype, designated here: BM! [BM000565932]; isolectotypes: K! [K000867593, K000867594], CAL).
Phyllagathis fruticosa (Ridl.) C.Hansen ex Cellin., Blumea 47(3): 473. 2002.

#### Notes.

When publishing *A.fruticosus*, Ridley (1908) designated L.Wray and H.C.Robinson 5453 as the type without citing a particular herbarium, only stating that the whole collection made by Robinson’s expedition should be sent to the British Museum (BM). Nayar revised *Perilimnastes* in 1974 and noted the specimen in BM as holotype of this species. This was probably only a speculation rather than deliberate lectotypification. In the revision of *Phyllagathis*, Cellinese (2002) chose a duplicate sheet in K as the lectotype, but did not include the phrase “designated here” in the typification statement, as required by Art. 7.11 of the Code (Turland et al. 2018). The specimen sheet in BM [BM000565932] is here designated as the lectotype to eliminate any uncertainty.

### ﻿ Perilimnastes guillauminii

Taxon classificationPlantaeMyrtalesMelastomataceae

(H.L.Li) Ying Liu comb. nov.

407117CA-023B-56A7-80DC-774AD604F07D

urn:lsid:ipni.org:names:77335510-1

Phyllagathis guillauminii H.L.Li, J. Arnold Arbor. 25: 29, in obs. 1944 (Basionym). Type: Cochinchine. Bien Hoa, Bao Chiang, L.Pierre s.n. (lectotype, designated by Hansen [1992]: P! [P05200250], drawing, C! [C10014976]). Additional syntype: Vietnam. Annam: Hue, s.n. (P! [P05200249]).
Phyllagathis hirsuta Guillaumin, Notul. Syst. (Paris) 2: 325, 1913, non Cogn. (1894).

### ﻿ Perilimnastes melastomatoides

Taxon classificationPlantaeMyrtalesMelastomataceae

(Merr. & Chun) Ying Liu comb. nov.

95153F93-152C-5178-8357-6D0A891FE5B0

urn:lsid:ipni.org:names:77335511-1

Osbeckia melastomatoides Merr. & Chun, Sunyatsenia 2: 293. 1935 (Basionym). Type: China. Hainan: Mo San Leng, 21 Nov 1932, N.K.Chun and C.L.Tso 44310 (lectotype, designated by Li [1944]: A! [A00055333]; isolectotypes: NY! [NY00229583], US! [US00120468]).
Phyllagathis melastomatoides (Merr. & Chun) W.C.Ko, Acta Phytotax. Sin. 8(3): 267. 1963.

### ﻿ Perilimnastes melastomatoides var. brevipes

Taxon classificationPlantaeMyrtalesMelastomataceae

(W.C.Ko) Ying Liu comb. nov.

2782811A-2A02-5C70-8C08-00F9DBBA3034

urn:lsid:ipni.org:names:77335512-1

Phyllagathis melastomatoides var. brevipes W.C.Ko, Acta Phytotax. Sin. 8(3): 268. 1963 (Basionym). Type: China. Hainan: Ya Hsien, Yulinwan, 15 Nov 1933, C.Wang 35035 (holotype: HC; isotypes: IBK! [IBK00129997], IBSC! [IBSC0246912, IBSC0003951], NY! [NY00079855]).

### ﻿ Perilimnastes multisepala

Taxon classificationPlantaeMyrtalesMelastomataceae

J.H.Dai, T.V.Do & Ying Liu, PhytoKeys 235: 4. 2023.

E7E7312C-FBA8-538F-A3E3-5BCFC7D507B7

#### Type.

Vietnam. Quảng Nam Province: Đại Lộc, about 400 m south of Khu Du Lich Sinh Thai Khe Lim, along newly opened road, 574 m elevation, on rocks along a stream, 23 Nov 2019, Jin-hong Dai and Ying Liu 821 (holotype: PE; isotypes: A, SYS, VNMN).

### ﻿ Perilimnastes ovalifolia

Taxon classificationPlantaeMyrtalesMelastomataceae

(H.L.Li) Ying Liu comb. nov.

B97F560C-32F6-5FA0-8DAE-674EF2F3A14C

urn:lsid:ipni.org:names:77335513-1

Phyllagathis ovalifolia H.L.Li, J. Arnold Arbor. 25: 31. 1944 (Basionym). Type: China. Yunnan: Ping-pien Hsien, 1,400 m, 7 Aug 1934, Tsai 61456 (holotype: A! [A00055329]; isotypes: PE! [PE00781713, PE00781714]).
Phyllagathis calisaurea C.Chen, Bull. Bot. Res., Harbin 4(3): 45. 1984. Type: China. Guangxi: Jingxi, Nanpo, Diding, 20 Jun 1978, T. Fang and X. H. Lu 23672 (holotype: GXMI! [GXMI050227]; isotype: GXMI! [GXMI050228]).
Phyllagathis ovalifolia var. pauciflora R.H.Miao, Acta Sci. Nat. Univ. Sunyatseni 32(4): 61. 1993. Type: China. Yunnan: Maguan County, Z.R.Xu and B.Li GL86-7974 (holotype: SYS! [SYS00103897]).

#### Notes.

*Phyllagathiscalisaurea* was described, based on specimens collected in western Guangxi, China (Chen 1984b). Subsequent authors did not recognise it as a distinct species and synonymised it within *P.ovalifolia* (Hansen 1992; Chen and Renner 2007). *Phyllagathiscalisaurea* and *P.ovalifolia* have adjacent distribution ranges (Guangxi vs. Yunnan, China). They are morphologically quite similar, with the only differences being leaf size (6.5–11.5 × 2–3.7 cm vs. 9–18 × 3–8.5 cm), leaf shape (ovate lanceolate vs. ovate to elliptic) and indumentum of the stems and leaves. Nonetheless, they failed to form a monophyletic group in both the previous (Zhou et al. 2022) and current phylogenetic analyses (Fig. 1). As only one accession of *P.ovalifolia* was included in these analyses, the boundary between *P.ovalifolia* and *P.calisaurea* needs to be further investigated using multiple accessions from across the distribution range. For the time being, we adhere to the species delimitation proposed by Hansen (1992) and Chen and Renner (2007).

### ﻿ Perilimnastes rupicola

Taxon classificationPlantaeMyrtalesMelastomataceae

M.P.Nayar, J. Bombay Nat. Hist. Soc. 71(1): 173. 1974.

91A48B1B-796A-5EFA-905F-65DF87B42CE8

Anerincleistus rupicola (M.P.Nayar) J.F.Maxwell, Gard. Bull. Singapore 35(2): 215. 1983.
Phyllagathis rupicola (M.P.Nayar) C.Hansen ex Cellin., Blumea 48(1): 92. 2003.

#### Type.

Malaysia. Sarawak: Mt Dulit, Ulu Koyan, alt. 800 m, 16 Sept 1932, S.Synge 503 (holotype: K! [K000867704]).

### ﻿ Perilimnastes sessilifolia

Taxon classificationPlantaeMyrtalesMelastomataceae

(C.Hansen) Ying Liu comb. nov.

FFBBD98A-34FD-5427-9453-7C4AB9BE0271

urn:lsid:ipni.org:names:77335514-1

Phyllagathis sessilifolia C.Hansen, Bull. Mus. Natl. Hist. Nat., B, Adansonia Sér. 4, 12(1): 39. 1990 (Basionym). Type: Indochine. Annam: Nui Bach Ma station d’altitude de Huê, 6 Sept 1938, E.Poilane 27614 (holotype: P! [P02274752]; isotypes: P! [P02274753, P02274754]).

### ﻿ Perilimnastes setipetiola

Taxon classificationPlantaeMyrtalesMelastomataceae

J.H.Dai, T.V.Do & Ying Liu, PhytoKeys 235: 5. 2023.

FDBA2C74-8C5C-54DB-9A37-313A1AC9A329

#### Type.

Vietnam. Lâm Đồng Province: Đà Lạt, Bidoup Nui Ba National Park, 1,500–1,700 m elevation, at damp places under forest, 29 Nov 2019, Jin-hong Dai and Ying Liu 836 (holotype: PE; isotypes: A, SYS, VNMN).

### ﻿ Perilimnastes setotheca

Taxon classificationPlantaeMyrtalesMelastomataceae

(H.L.Li) Ying Liu comb. nov.

283804F4-9944-5571-817D-E7C455728670

urn:lsid:ipni.org:names:77335515-1

Phyllagathis setotheca H.L.Li, J. Arnold Arbor. 25: 32. 1944 (Basionym). Type: China. Guangxi: Shih Wan Tai Shan, 21 Jul 1937, H.Y.Liang 69817 (holotype: A! [A00055328]; isotypes: IBK! [IBK00127588], IBSC! [IBSC0003958], PE! [PE00781748]).

### ﻿ Perilimnastes setotheca var. setotuba

Taxon classificationPlantaeMyrtalesMelastomataceae

(C.Chen) Ying Liu comb. nov.

DFACA701-3BFF-5FD0-8696-5244F9ACDB0F

urn:lsid:ipni.org:names:77335516-1

Phyllagathis setotheca var. setotuba C.Chen, Bull. Bot. Res., Harbin 4(3): 44. 1984 (Basionym). Type: China. Guangdong: Yangjiang, Longgaoshan, 29 May 1956, Wang 41508 (holotype: IBSC! [IBSC0003999]; isotype: IBK! [IBK00127590]).

### ﻿ Perilimnastes stenophylla

Taxon classificationPlantaeMyrtalesMelastomataceae

(Merr. & Chun) Ying Liu comb. nov.

E4B71489-3403-5F63-A0CF-44F15573164A

urn:lsid:ipni.org:names:77335517-1

Bredia stenophylla Merr. & Chun, Sunyatsenia 5: 146. 1940 (Basionym). Type: China. Hainan: Yaichow, 11 Aug 1933, Liang 62530 (lectotype, designated by Li [1944]: A! [A00055335]; isolectotypes: E! [E00090770], G! [G00353917], NY! [ny00221474]).
Phyllagathis stenophylla (Merr. & Chun) H.L.Li, J. Arnold Arbor. 25: 32. 1944.

### ﻿ Perilimnastes suberalata

Taxon classificationPlantaeMyrtalesMelastomataceae

(C.Hansen) Ying Liu comb. nov.

6D7F7AC4-71FF-5552-AF46-C3A3FB55613B

urn:lsid:ipni.org:names:77335518-1

Phyllagathis suberalata C.Hansen, Bull. Mus. Natl. Hist. Nat., B, Adansonia Sér. 4, 12(1): 39. 1990 (Basionym). Type: Indochine. Annam: Nui Bach Ma station près de Huê Grande Cascade, 16 Apr 1939, E.Poilane 29758 (holotype: P! [P02274749]; isotypes: P! [P02274750, P02274751]).

### ﻿ Perilimnastes ternata

Taxon classificationPlantaeMyrtalesMelastomataceae

(C.Chen) Ying Liu comb. nov.

5A62CC1A-6815-514B-B408-88CE0E42A121

urn:lsid:ipni.org:names:77335519-1

Phyllagathis ternata C.Chen, Bull. Bot. Res., Harbin 4(3): 49. 1984 (Basionym). Type: China. Guangdong: Xinyi, Dadufoshan, stream side, 10 Aug 1931, S.P.Ko 51772 (holotype: IBSC! [IBSC0004000]; isotype: IBSC! [IBSC0223824]).
Phyllagathis xinyiensis Z.J.Feng, J. South China Agr. Univ. 15(4): 75. 1994. Type: China. Guangdong: Xinyi, Dawuling, infra silvis, Z.J.Feng 53621 (holotype: CANT).

### ﻿ Perilimnastes uniflora

Taxon classificationPlantaeMyrtalesMelastomataceae

J.H.Dai, T.V.Do & Ying Liu, PhytoKeys 235: 11. 2023.

2F0D004A-E35F-50C3-89E8-6267405A72BD

#### Type.

Vietnam. Đà Nẵng: Hòa Ninh, Ba Na Hills, 1,360 m elevation, in forests on damp rocks along steam, 22 Nov 2019, Jin-hong Dai and Ying Liu 814 (holotype: PE; isotypes: A, SYS, VNMN).

### ﻿ Perilimnastes nana

Taxon classificationPlantaeMyrtalesMelastomataceae

C.Y.Zou & Ying Liu sp. nov.

42EB1963-7A5C-5E18-84F6-B94DF1F679B9

urn:lsid:ipni.org:names:77335520-1

Figs 6 , 7

#### Type.

China. Guangdong Province: Taishan County, Chixi Town, near Zhuxing Village, 200–300 m elevation, amongst rocks along a stream in forests, 15 Jun 2022, *Chun-yu Zou* 3608 (holotype: IBK; isotypes: IBK, PE).

**Figure 6.**
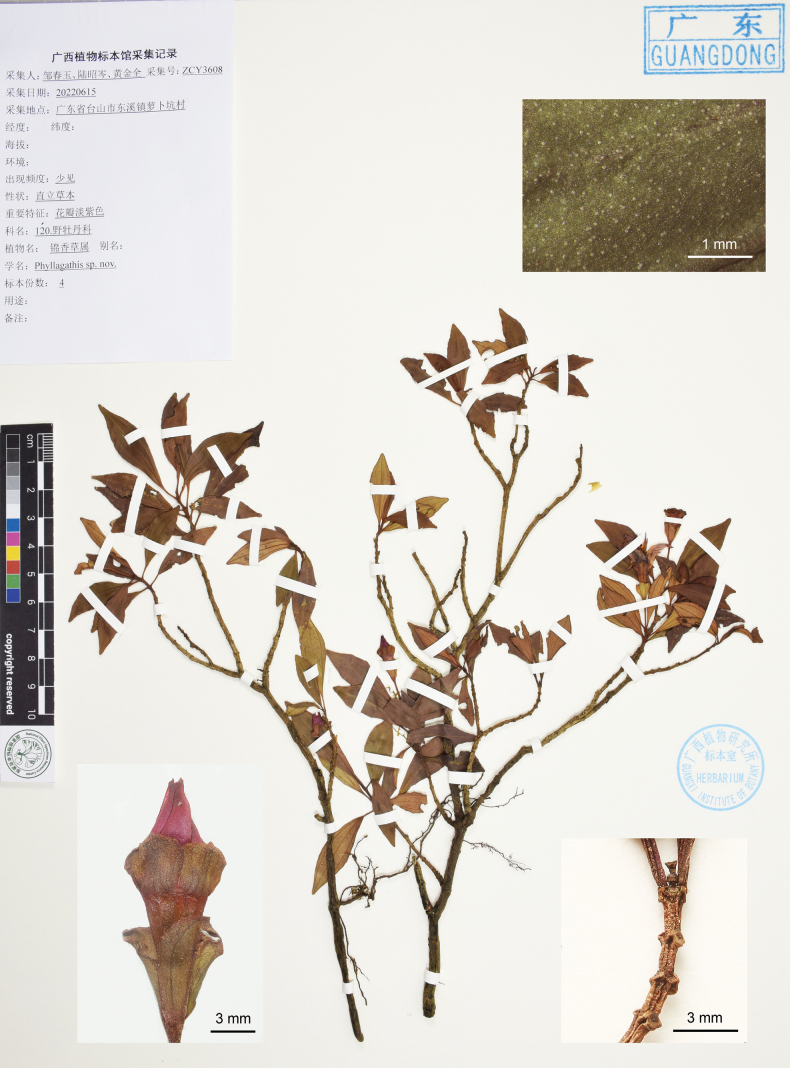
Holotype of *Perilimnastesnana*, Chun-yu Zou 3608 (IBK). The insets show details of leaf surface under stereoscope, branchlet and flower. Scale bars: 10 cm, 1 mm (upper right inset); 3 mm (lower insets).

#### Diagnosis.

Resembles *P.stenophylla* in leaf morphology, but differs in height (to 0.15 m vs. 0.8 m tall), number of flowers per inflorescence (1 vs. 2–3-flowered), length of the peduncle (10–22 mm vs. 4 mm) and the shape of calyx lobes (broadly obovate vs. narrowly triangular). Resembles *P.setotheca* in having 4-sided branchlets, large and persistent bracts below flower and stamen morphology, but differs in plant size (to 0.15 m vs. 1 m tall), leaf shape and size (oblong-lanceolate or obovate-lanceolate, 1.7–7 × 0.73–2.2 cm vs. oblong-lanceolate, elliptic or obovate, 10–20 × 3–8 cm) and number of flowers per inflorescence (1-flowered vs. 3 to more than 20-flowered).

**Figure 7.**
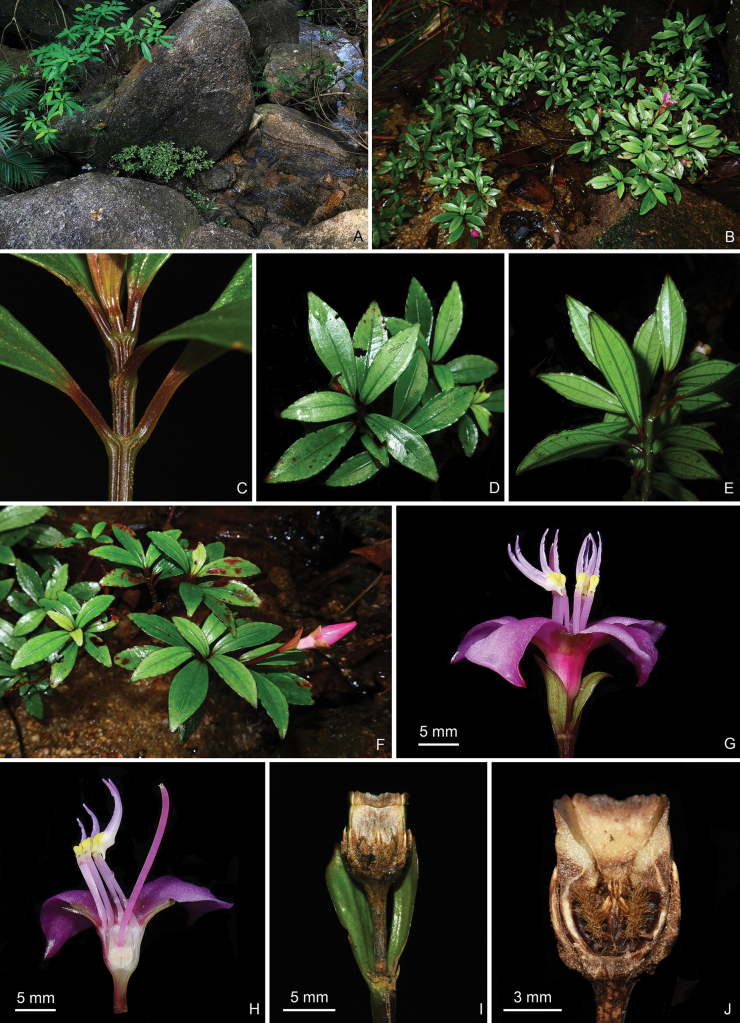
*Perilimnastesnana***A** habitat **B** habit **C** close-up of a branchlet **D** adaxial leaf surfaces **E** abaxial leaf surfaces **F** a flowering branch showing an inflorescence with a single flower and two large bracts **G** lateral view of a flower **H** longitudinal section of a flower showing stamen morphology **I** lateral view of an old capsule with one persistent bract removed **J** longitudinal section of an old capsule showing enlarged ovary crown and morphology of the placental column and placentas. Scale bars: 5 mm (**G–I**); 3 mm (**J**). All from Chun-yu Zou 3608 (IBK, PE).

#### Description.

Dwarf shrubs, much-branched, ascending, to 0.15 m tall, with druses in many parts. Stems and leaves sparsely puberulent with minute brown hairs (with few-celled stalk and a glandular head) when young, glabrous when mature. Stems obtusely 4-sided; branchlets 4-sided, with four ribs and two additional ridges extending from the base of the leaf petioles. Leaves opposite, equal to subequal in a pair, glabrous when mature; petiole 2–22 mm; leaf blade oblong-lanceolate or obovate-lanceolate, 1.7–7 × 0.73–2.2 cm, thick papery, 3-veined, green to dark green adaxially, pale green abaxially, base cuneate, apex acute, margin basally entire and remotely denticulate to repand above the base or the middle. Inflorescences terminal, peduncles 1–2.2 cm long; flower solitary, subtended by one or two pairs of leaf-like bracts, bracts 1–1.8 × 0.7–0.9 cm, persistent in fruit. Flowers 4-merous; pedicel 4-sided, ca. 4 mm long in flower and 4–10 mm in fruit; hypanthia funnel-shaped, glabrous, ca. 7 × 6 mm; calyx lobes 4, broadly obovate, glabrous, 4–5 × 5 mm; petals pinkish-purple, ca. 15 × 7 mm, obovate, oblique, apex acute or short acuminate, glabrous on both sides; stamens 8, isomorphic, filaments 8–10 mm long, white or pink, glabrous, anthers ovate-lanceolate, curved to ventral side, pinkish-purple with yellow base, ca. 9 mm long, connective dorsally forming a 0.7–1 mm long spur and ventrally forming two yellow ridges; ovary ca. 3 mm long, half as long as hypanthium (crown excluded), ovary crown wedge-like, 4-lobed; styles 20 mm long. Old (post-mature) capsules cup-shaped, 7–9 × 4–7 mm, 4-sided; hypanthium 8-ribbed; crown enlarged and enclosing an obpyramidal space; placental column unbeaked, 4-horned; placenta thready.

#### Phenology.

Flowers in May and June, old capsules in October.

#### Etymology.

The specific epithet is based on the habit of this species, viz. dwarf shrubs to 15 cm tall.

#### Distribution.

**Figure 8.**
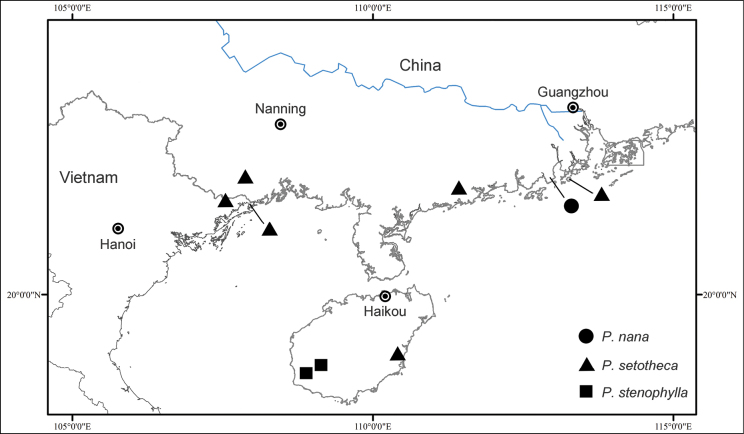
Distribution of *Perilimnastesnana* (solid circle), *P.setotheca* (triangle) and *P.stenophylla* (square).

*Perilimnastesnana* is currently known from Taishan County, Guangdong Province, China (Fig. 8). It grows amongst rocks along streams in the forest, at 200–300 m elevation.

#### Notes.

During a survey of herbarium specimens of *Phyllagathis* in IBSC, a collection (Ze-xian Li et al. 516) from Taishan, Guangdong, China caught our attention. This plant (*P.nana*) closely resembles *P.stenophylla* from Hainan Island in the oblong-lanceolate leaf blades and was misidentified as the latter species. Closer inspection reveals that it has strictly 1-flowered inflorescences and broadly obovate calyx lobes, which distinguishes it from *P.stenophylla*. Field trips in 2022 and 2023 revealed other differences between the two species, such as plant size and peduncle length. *Perilimnastesnana* is phylogenetically closest to *P.setotheca*, a species found in Guangdong, Guangxi and Hainan China (Fig. 8). However, they differ markedly in plant size, leaf shape and size and number of flowers per inflorescence. As a result, *P.nana* is quite distinct from its closest relatives, prompting us to describe it as a new species.

#### Additional specimen examined.

China. Guangdong Province: Taishan County, Chixi Town, Zhuxing Village, 220 m elevation, 17 Oct 2023, Ying Liu 892 (SYS); Taishan County, Chixi Town, Liugushan, 8 May 1981, Ze-xian Li et al. 516 [IBSC (IBSC0223903)].

### ﻿Key to the species of *Perilimnastes*

**Table:** 

1	Raphides present, appearing on leaf surfaces as whitish oblong spots when dried	**2**
–	Raphides absent	**13**
2	Flowers always solitary	**3**
–	Flowers often in few-flowered umbels, sometimes or rarely reduced to a solitary flower, rarely many-flowered	**4**
3	With sparse minute brown glands on branchlets and leaves when young, glabrescent; leaf blade obovate to obovate-lanceolate, base cuneate to narrowly cuneate	***P.uniflora***
–	With uniseriate, pale brown hyaline hairs on branchlets and leaves; leaf blade elliptic, base acute to rounded	***P.dispar***
4	Leaf blades broadly obovate to suborbicular, 2.5–3.5 cm long	***P.brookei***
–	Leaf blades ovate, elliptic, narrowly elliptic, or elliptic-lanceolate, often > 4 cm long	**5**
5	Inflorescence sessile or nearly sessile	**6**
–	Inflorescence with 1–3.5 cm long peduncles	**10**
6	Leaf blades narrowly elliptic	**7**
–	Leaf blades broadly elliptic or elliptic	**8**
7	Hypanthia with sparse minute brown glands; anthers yellow	***P.rupicola***
–	Hypanthia with sparse minute brown glands and dense patent brown bristles; anthers purplish	***P.guillauminii***
8	Petioles densely villous with appressed, brown hyaline hairs, without bristles	***P.banaensis***
–	Petioles with bristles	**9**
9	Mature stem with curly retrorse bristles; leaf bases rounded to broadly rounded; anthers yellowish	***P.elliptica***
–	Mature stem glabrescent; leaf bases cuneate; anthers pinkish	***P.setipetiola***
10	Stems hirsute with crooked, multiseriate hairs	**11**
–	Stems hirsute with straight, multiseriate hairs	**12**
11	Leaf blade oblanceolate to elliptic-lanceolate, 4.8–14 × 1.1–2.7 cm; peduncle pubescent with minute, appressed hairs	***P.elegans***
–	Leaf blade elliptic to long elliptic, 5–13 × 1.5–4 cm; peduncle pubescent with spreading hairs	***P.deltoidea***
12	Stems retrorse hirsute with multiseriate hairs or pubescent with hyaline uniseriate hairs; leaf blade 7–18 × (2–)3–8.5 cm	***P.ovalifolia***
–	Stems densely setose with multiseriate hairs; leaf blade 5–8 × 2.5–4 cm	***P.ternata***
13	Leaf base broadly cordate	***P.sessilifolia***
–	Leaf base broadly cuneate, cuneate, or acuminate	**14**
14	Mature stems and leaves with appressed or ascending bristles	***P.melastomatoides***
–	Mature stems and leaves glabrous	**15**
15	Leaves unequal, rarely subequal, in a pair	***P.suberalata***
–	Leaves usually equal or subequal in a pair	**16**
16	Leaf blades 10–20 × 3–8 cm; inflorescences subtended by an involucre of several bracts (often 4)	***P.setotheca***
–	Leaf blades 2.8–10(–14) × 0.6–2.4(–4.2) cm; inflorescences subtended by a pair of small leaves/bracts	**17**
17	Calyx lobes 4–8	***P.multisepala***
–	Calyx lobes 4	**18**
18	Dwarf shrubs to 15 cm tall; inflorescence 1-flowered	***P.nana***
–	Shrubs to 80–100 cm tall; inflorescence 1–4-flowered	**19**
19	Calyx lobes ca. 3 mm long, not keeled; anthers purplish	***P.stenophylla***
–	Calyx lobes 4–7 mm long, keeled; anthers yellow	***P.fruticosa***

## Supplementary Material

XML Treatment for Perilimnastes

XML Treatment for Perilimnastes banaensis

XML Treatment for Perilimnastes brookei

XML Treatment for Perilimnastes deltoidea

XML Treatment for Perilimnastes dispar

XML Treatment for Perilimnastes elegans

XML Treatment for Perilimnastes elliptica

XML Treatment for Perilimnastes fruticosa

XML Treatment for Perilimnastes guillauminii

XML Treatment for Perilimnastes melastomatoides

XML Treatment for Perilimnastes melastomatoides var. brevipes

XML Treatment for Perilimnastes multisepala

XML Treatment for Perilimnastes ovalifolia

XML Treatment for Perilimnastes rupicola

XML Treatment for Perilimnastes sessilifolia

XML Treatment for Perilimnastes setipetiola

XML Treatment for Perilimnastes setotheca

XML Treatment for Perilimnastes setotheca var. setotuba

XML Treatment for Perilimnastes stenophylla

XML Treatment for Perilimnastes suberalata

XML Treatment for Perilimnastes ternata

XML Treatment for Perilimnastes uniflora

XML Treatment for Perilimnastes nana

## ﻿Additional information

### Author ORCIDs

Ying Liu https://orcid.org/0000-0003-0613-837X

Jin-Hong Dai https://orcid.org/0000-0001-5069-6016

Qi-Yuan Zhuang https://orcid.org/0000-0003-2025-6487

Chun-Yu Zou https://orcid.org/0000-0001-6004-6551

Kai-Nan Ma https://orcid.org/0009-0008-2381-939X

### Data availability

All of the data that support the findings of this study are available in the main text or Supplementary Information.

## Supplementary materials

Supplementary material 1 Source of materials studied Data type xlsx Explanation note The name of the new species is indicated in bold. https://binary.pensoft.net/file/977231This dataset is made available under the Open Database License (http://opendatacommons.org/licenses/odbl/1.0/). The Open Database License (ODbL) is a license agreement intended to allow users to freely share, modify, and use this Dataset while maintaining this same freedom for others, provided that the original source and author(s) are credited.Ying Liu, Jin-Hong Dai, Qi-Yuan Zhuang, Chun-Yu Zou, Kai-Nan Ma

## References

[B1] Cellinese N (2002) Revision of the genus *Phyllagathis* Blume (Melastomataceae: Sonerileae) I. The species of Burma, Thailand, Peninsular Malaysia and Sumatra. Blumea 47: 463–492.

[B2] Cellinese N (2003) Revision of the genus *Phyllagathis* (Melastomataceae: Sonerileae) II. The species in Borneo and Natuna Island. Blumea 48(1): 69–97. 10.3767/000651903X686060

[B3] Chen C (1984a) Melastomataceae. In: Chen C (Ed.) Flora Reipublicae Popularis Sinicae, vol 53.Science Press, Beijing, 135–293.

[B4] Chen C (1984b) Materia ad flora Melastomataceae sinensium. Bulletin of Botanical Research 4: 33–68.

[B5] Chen C Renner SS (2007) Melastomataceae. In: Wu ZY Raven PH Hong DY (Eds) Flora of China, vol.13. Science Press, Beijing; Missouri Botanical Garden Press, St. Louis, 360–399.

[B6] Danecek P Auton A Abecasis G Albers CA Banks E DePristo MA Handsaker RE Lunter G Marth G Sherry ST McVean G Durbin R (2011) The variant call format and VCFtools. Bioinformatics (Oxford, England) 27(15): 2156–2158. 10.1093/bioinformatics/btr330 21653522 PMC3137218

[B7] Hansen C (1992) The genus *Phyllagathis* (Melastomataceae): Characteristics; delimitation; the species in Indo-China and China. Bulletin du Museum National d’Histoire Naturelle. Section B, Adansonia, Botanique. Phytochimie 14: 355–428.

[B8] Kalyaanamoorthy S Minh BQ Wong TKF Von Haeseler A Jermiin LS (2017) ModelFinder: Fast model selection for accurate phylogenetic estimates. Nature Methods 14(6): 587–589. 10.1038/nmeth.4285 28481363 PMC5453245

[B9] Li HL (1944) Studies in the Melastomataceae of China. Journal of the Arnold Arboretum 25(1): 1–42. 10.5962/p.172676

[B10] Li H Durbin R (2010) Fast and accurate long-read alignment with Burrows-Wheeler transform. Bioinformatics (Oxford, England) 26(5): 589–595. 10.1093/bioinformatics/btp698 20080505 PMC2828108

[B11] Liu Y Veranso-Libalah MC Kadereit G Zhou RC Quakenbush JP Lin CW Wai JS (2022) Systematics of the Tribe Sonerileae. In: Goldenberg R Michelangeli FA Almeda F (Eds) Systematics, Evolution, and Ecology of Melastomataceae.Springer Nature, Cham, Switzerland, 321–343. 10.1007/978-3-030-99742-7_15

[B12] Maxwell JF (1982) Taxonomic and nomenclatural notes on *Oxyspora* DC., *Anerincleistus* Korth., *Poikilogyne* Baker f., and *Allomorphia* BL. (Melastomataceae, tribe Oxysporeae). Gardens’ Bulletin (Singapore) 35(2): 209–226.

[B13] Maxwell JF (1989) The genus *Anerincleistus* Korth. (Melastomataceae). Proceedings of the Academy of Natural Sciences of Philadelphia 141: 29–72.

[B14] McKenna A Hanna M Banks E Sivachenko A Cibulskis K Kernytsky A Garimella K Altshuler D Gabriel S Daly M DePristo MA (2010) The genome analysis toolkit: A mapreduce framework for analyzing next-generation DNA sequencing data. Genome Research 20(9): 1297–1303. 10.1101/gr.107524.110 20644199 PMC2928508

[B15] Nayar MP (1974) A synopsis of the genus *Periilimnastes* Ridley (Melastomataceae). Journal of the Bombay Natural History Society 71: 172–175.

[B16] Nguyen L Schmidt HA von Haeseler A Minh BQ (2015) IQ-TREE: A fast and effective stochastic algorithm for estimating maximum likelihood phylogenies. Molecular Biology and Evolution 32(1): 268–274. 10.1093/molbev/msu300 25371430 PMC4271533

[B17] Purcell S Neale BM Toddbrown K Thomas L Ferreira MA Bender D Maller J Sklar P De Bakker PIW Daly MJ Sham PC (2007) PLINK: A tool set for whole-genome association and population-based linkage analyses. The American Journal of Human Genetics 81(3): 559–575. 10.1086/519795 17701901 PMC1950838

[B18] Ridley HN (1908) On a collection of plants made by H.C. Robinson and L. Wray from Gunong Tahan, Pahang. Botanycal Journal of the Linnean Society of London 38(266): 301–336. 10.1111/j.1095-8339.1908.tb02454.x

[B19] Ridley HN (1918) New and rare Malayan Plants Series X. Journal of the Straits Branch of the Royal Asiatic Society 79: 63–100.

[B20] Ridley HN (1922) Melastomataceae. In: Ridley HN (Ed.) The flora of the Malay Peninsula, vol.1. L. Reeve and Company, Limited, London, 760–819. 10.5962/bhl.title.10921

[B21] Turland NJ Wiersema JH Barrie FR Greuter W Hawksworth DL Herendeen PS Knapp S Kusber WH Li DZ Marhold K May TW McNeill J Monro AM Prado J Price MJ Smith GF (2018) International Code of Nomenclature for algae, fungi, and plants (Shenzhen Code) adopted by the Nineteenth International Botanical Congress Shenzhen, China, July 2017. Regnum Vegetabile 159. Koeltz Botanical Books, Glashütten. 10.12705/Code.2018

[B22] Zeng SJ Zou LH Wang P Hong WJ Zhang GQ Chen LJ Zhuang XY (2016) Preliminary phylogeny of *Fordiophyton* (Melastomataceae), with the description of two new species. Phytotaxa 247(1): 45–61. 10.11646/phytotaxa.247.1.3

[B23] Zhou RC Zhou QJ Liu Y (2018) *Brediarepens*, a new species from Hunan, China. Systematic Botany 43(2): 544–551. 10.1600/036364418X697265

[B24] Zhou QJ Lin CW Dai JH Zhou RC Liu Y (2019a) Exploring the generic delimitation of *Phyllagathis* and *Bredia* (Melastomataceae): A combined nuclear and chloroplast DNA analysis. Journal of Systematics and Evolution 57(3): 256–267. 10.1111/jse.12451

[B25] Zhou QJ Dai JH Lin CW Denda T Zhou RC Liu Y (2019b) Recircumscription of *Bredia* and resurrection of *Tashiroea* (Sonerileae, Melastomataceae) with description of a new species *T.villosa*. PhytoKeys 127: 121–150. 10.3897/phytokeys.127.36608 31379453 PMC6661266

[B26] Zhou QJ Lin CW Ng WL Dai JH Denda T Zhou RC Liu Y (2019c) Analyses of plastome sequences improve phylogenetic resolution and provide new insight into the evolutionary history of Asian Sonerileae/Dissochaeteae. Frontiers in Plant Science 10: 1477. 10.3389/fpls.2019.01477 PMC6881482 31824528

[B27] Zhou QJ Dai JH Lin CW Ng WL Van Do T Wai JS Michelangeli FA Reginato M Zhou RC Liu Y (2022) Out of chaos: Phylogenomics of Asian Sonerileae. Molecular Phylogenetics and Evolution 175: 107581. 10.1016/j.ympev.2022.107581 35810973

